# Neural mechanisms underlying the after-effects of repetitive paired-pulse TMS with β tACS on the human primary motor cortex

**DOI:** 10.1038/s41598-025-92444-4

**Published:** 2025-03-01

**Authors:** Hisato Nakazono, Katsuya Ogata, Tsubasa Mitsutake, Akinori Takeda, Emi Yamada, Shozo Tobimatsu

**Affiliations:** 1 Department of Occupational Therapy, Faculty of Medical Science, Fukuoka International University of Health and Welfare, Fukuoka, 814-0001 Japan; 2 Department of Health Sciences, Graduate School of Medical Sciences, Kyushu University, Fukuoka, 812-8582 Japan; 3 Department of Pharmaceutical Sciences, School of Pharmacy at Fukuoka, International University of Health and Welfare, Fukuoka, 831-8501 Japan; 4 Clinical Research Center, Saga University Hospital, Saga, 849-8501 Japan; 5 Research Center for Brain Communication, Research Institute, Kochi University of Technology, Kochi, 782-8502 Japan; 6 Department of Linguistics, Faculty of Humanities, Kyushu University, Fukuoka, 819-0395 Japan; 7 Department of Orthoptics, Faculty of Medical Science, Fukuoka International University of Health and Welfare, Fukuoka, 814-0001 Japan

**Keywords:** Transcranial alternating current stimulation, Repetitive paired-pulse transcranial magnetic stimulation, Combined stimulation, Paired-pulse paradigm, Phase dependence, Primary motor cortex, Neuroscience, Motor cortex

## Abstract

We previously reported that repetitive paired-pulse transcranial magnetic stimulation (TMS; rPPS) synchronized to the peak phase of transcranial alternating current stimulation (tACS) at the β frequency induced long-lasting after-effects on primary motor cortex (M1) with less inter-individual variability compared with rPPS alone. Here, we investigated the plasticity mechanisms underlying combined stimulation effects using paired-pulse TMS paradigms. rPPS was applied to the peak phase of β tACS (rPPS-tACS-peak) or sham tACS (rPPS alone), or tACS was delivered without rPPS (tACS alone). Resting motor threshold (RMT) and motor evoked potentials (MEPs) elicited by single-pulse TMS, short-interval intracortical inhibition (SICI), intracortical facilitation (ICF), short-latency afferent inhibition (SAI), and short-interval intracortical facilitation (SICF) were measured before and after intervention. rPPS-tACS-peak stimulation significantly increased MEPs compared with other conditions after intervention. Although I-wave interaction was expected to be produced by the facilitation effect of rPPS, rPPS-tACS-peak did not change SICF. In contrast, SAI was decreased in rPPS-tACS-peak compared with baseline. In the control experiment, rPPS-tACS-trough did not change MEPs, SAI, and SICF. Therefore, the after-effects of rPPS-tACS-peak on M1 may be caused by a partial reduction in the inhibitory circuit mediated by cholinergic interneurons, rather than an enhancement of the facilitatory effects of rPPS.

## Introduction

Cortical oscillations reflect the network activity that is generated in specific or large-scale neuronal networks, which represents an essential component of information processing in the brain^1^. Brain states change in the time scale of milliseconds to seconds because the temporospatial dynamics of spontaneous network activity influence neuronal activity^1,2^. Therefore, the phase information of specific brain oscillation can reflect states of heightened excitability in the brain cortex^3^. Moreover, neural oscillations may play a causal role in neural communication, and specific abnormal oscillatory patterns can lead to the neurocognitive changes observed during aging, and in neurological and psychiatric disorders^4^. Thus, there is increasing interest in tools for externally modulating neural oscillations for research and therapy^5^. Transcranial alternating current stimulation (tACS) is a non-invasive brain stimulation (NIBS) technique for entraining ongoing cortical oscillations and modulating cortical neural activity in a frequency-dependent manner^6–8^. In the primary motor cortex (M1), tACS in the β range (20 Hz) has been reported to increase motor evoked potential (MEP) amplitudes during stimulation^9,10^. Subsequent studies have suggested that this effect is dependent on the phase of tACS^11,12^.

Recently, combined stimulation of tACS and transcranial magnetic stimulation (TMS), has attracted attention as a novel technique to promote brain plasticity. Several studies have investigated the after-effects of combined stimulation of theta-burst stimulation (TBS) and tACS^13–15^. For example, tACS at γ (70 Hz) frequency combined with intermittent TBS (iTBS), which induces long-term potentiation (LTP)-like plasticity, increased M1 cortical excitability after stimulation compared with iTBS alone^14^. Additionally, our recent study revealed that β tACS modulated the LTP-like plasticity induced by repetitive paired-pulse TMS (rPPS, also known as iTMS) in a phase-dependent manner^16^. In rPPS, repetitive paired TMS at I-wave periodicity has been found to increase M1 cortical excitability after stimulation^17–20^. Combined stimulation, in which rPPS was adjusted to the peak phase of β tACS, increased MEP amplitudes for over 30 min after stimulation, while combined stimulation in the trough phase of β tACS abolished the facilitatory after-effects of rPPS. Meanwhile, combined stimulation of rPPS and α (10 Hz) tACS showed no phase-dependent effects^16^. These findings suggest that tACS may modulate LTP-like plasticity effects of rPPS in a phase- and frequency-dependent manner, but the fundamental mechanisms are largely unknown.

Several paired-pulse TMS paradigms have been developed to understand the mechanisms of M1 excitability, such as short-interval intracortical inhibition (SICI), intracortical facilitation (ICF), short-latency afferent inhibition (SAI) and short-interval intracortical facilitation (SICF). β tACS has been reported to modulate SICI, ICF, and SAI during stimulation^11,21^. SICI and ICF can be examined using paired-pulse TMS in which an initial subthreshold conditioning stimulus (CS) and a subsequent suprathreshold test stimulus (TS) are delivered at a 1–5 ms interstimulus interval (ISI) for SICI and a 7–20 ms ISI for ICF, respectively^22,23^. Whereas the CS leads to inhibition of MEP amplitudes elicited by TS in SICI, it leads to facilitation of MEP amplitudes elicited by TS in ICF. SICI measures intracortical inhibitory circuits, and is considered to reflect inhibition through gamma-aminobutyric acid-A (GABA_A_) receptors^24,25^. ICF tests the excitatory motor cortical circuits, and is thought to reflect glutamatergic N-methyl-D-aspartate (NMDA) facilitatory drive^26,27^. SAI refers to the MEP inhibition elicited by combining median nerve electrical stimulation and TMS of the motor cortex with an ISI of approximately the latency of the N20 component of somatosensory-evoked potential^28^. SAI can be used to explore sensorimotor integration^29^, and is considered to be a marker of cortical cholinergic activity in the cortex^30^.

Previous studies revealed that rPPS increased not only MEP amplitude but also SICF peaks after intervention^19^. Single-pulse TMS typically generates a brief train of high-frequency descending volleys at a periodicity of approximately 1.5 ms, known as indirect (I)-waves^31^. The SICF protocol consists of suprathreshold TMS followed by subthreshold TMS with an ISI of I-wave periodicity^32,33^. SICF is considered to represent an interaction between I-waves because the response to paired stimulation is facilitated if the second pulse is given at the peaks of the I-waves fired by the first pulse^32–34^.

As mentioned above, the techniques of SICI, ICF, SAI, and SICF can lead us to explore the detailed plasticity mechanisms of M1. To date, little is known about the neurophysiological mechanisms of the after-effects on M1 induced by rPPS combined with the peak phase of β tACS. Therefore, the aim of the current study was to elucidate the precise plasticity changes after intervention. To achieve this aim, we assessed the after-effects of combined rPPS with β tACS at peak phase (β tACS-peak), rPPS alone, and tACS alone using paired-pulse TMS paradigms (i.e., SICI, ICF, SAI, and SICF) in healthy human participants.

## Materials and methods

### Participants

Thirty-four subjects (20 women; mean age ± standard deviation [SD]: 21 ± 3.5 years) participated in this study. No participants had any history of neurological, psychiatric, or other medical problems. All participants were right-handed, according to the Edinburgh handedness inventory^35^. Written informed consent was obtained from each participant in accordance with the Declaration of Helsinki. This study was approved by the Ethics Committee of the International University of Health and Welfare (Approval Number: 22-fiuhw-003). The sample size was determined on the basis of our previous study of combined stimulation^16^. Eighteen subjects (eight women; 21.7 ± 4.9 years) completed the main experiment, while 17 participants (13 women; 20.3 ± 0.6 years; one of whom had also participated in the main experiment) enrolled in the control experiment.

### Transcranial magnetic stimulation

Single and paired-pulse TMS, and rPPS were performed using a DuoMAG MP-Quad TMS monophasic stimulator (DEYMED Diagnostic, Hronov, Czech Republic) connected to a figure-of-eight 70 mm air-cooled coil (70BF-Cool, DuoMAG). The coil was held tangentially to the scalp at an angle of 45° from the midline to induce a posterior-anterior current to the hand area of the left M1 hot spot. tACS electrodes were fixed to the scalp using plastic wrap with self-adhesive properties that made it easy to maintain the stability of the tACS electrodes. The left M1 hot spot was then re-identified. The position of the coil was marked with a pen on the plastic wrap to enable repositioning of the coil. The TMS parameters were determined after the tACS electrode was positioned over the M1 hotspot. Surface electromyography (EMG) was recorded from the right first dorsal interosseus (FDI) muscle using a pair of Ag-AgCl electrodes in a belly-tendon montage. The EMG signals were amplified using the Neuropack MEB-2200 (Nihon Kohden, Tokyo, Japan) with a band-pass filter of 10 Hz–2 kHz, digitized at a sampling rate of 10 kHz and stored in a computer using signal processing software (Multiscope PSTH, Medical Try System, Tokyo, Japan) for offline analysis. The analysis period was 300 ms in length, beginning 150 ms before TMS. Muscle relaxation was maintained online via visual feedback of EMG activity. Resting motor threshold (RMT) was defined as the lowest stimulus intensity necessary to evoke at least 50 µV MEPs in the relaxed muscle, in 50% of 10 consecutive trials. Active motor threshold (AMT) was defined as the lowest stimulus intensity that elicited at least 200 µV MEPs in 50% of 10 consecutive trials under slight voluntary contraction of the FDI muscle by approximately 10% of the maximal muscle strength. Participants maintained a slight contraction, viewing a raw EMG signal displayed on the LCD monitor. Single-pulse TMS was delivered at an intensity capable of eliciting approximately 1 mV MEPs in the relaxed muscle, and the intensity was maintained at a constant level throughout the experiment.

SICI, ICF, SICF, and SAI were studied using a paired-pulse technique, employing a conditioning-test design. For all paradigms, the TS was adjusted to elicit MEPs of approximately 1 mV amplitude (adjusted MEPs). The TS intensity was re-adjusted when necessary after intervention. SICI and ICF were determined by setting the CS at 90% AMT, employing an ISI of 3 ms for SICI and 10 ms for ICF^22,23^. To elicit SICF, the CS intensity was adjusted at 90% RMT, and delivering the CS after the TS, at ISIs of 1.5, 2.7 and 4.4 ms (SICF_1.5_, SICF_2.7_, and SICF_4.4_)^32,36^. SAI was examined by employing the CS of electrical rectangular pulses (0.2 ms) delivered to the right median nerve at the wrist, using a bipolar electrode with the cathode positioned proximally, at an intensity that evoked a slight thumb twitch. The individual N20 latency plus 3 ms was used as ISIs in SAI. Before the experiment, we recorded the somatosensory-evoked potentials by electrical stimulation of the median nerve at the right wrist (rate of stimulation 3 Hz, a total of 200 stimuli), and identified the latency of the N20 component. An active electrode was placed at C3’ (2 cm posterior to C3 in the international 10–20 system), and a reference electrode was placed at Fz^28^.

**Fig. 1:**
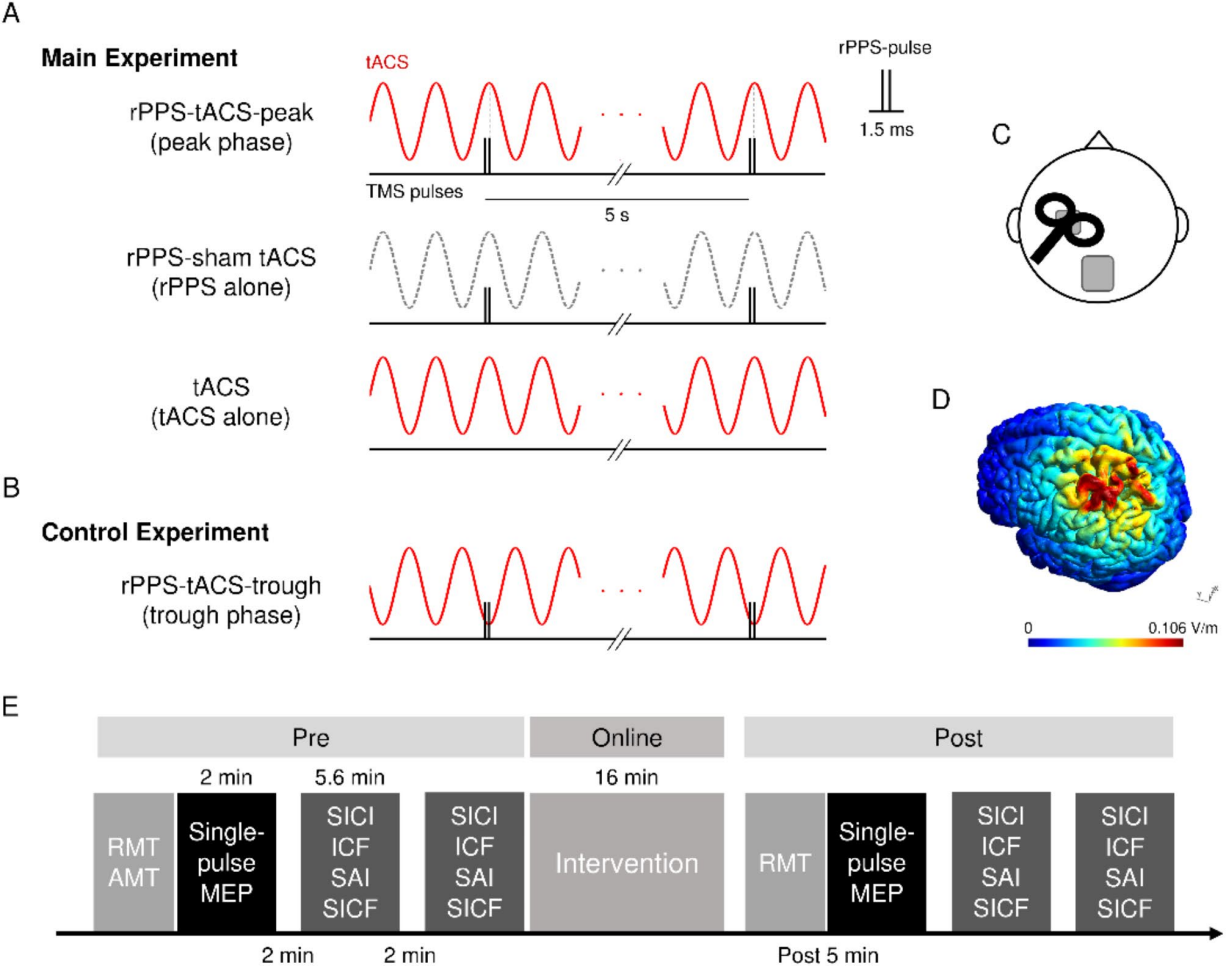
Experimental design. (**A**) Two conditions for the combined stimulation (left, upper and middle rows) and tACS alone conditions (lower row) in the main experiment. The second pulse of rPPS-pulse for rPPS adjusted to the tACS peak phase (90° phase) in the rPPS-tACS-peak condition. For the rPPS-sham tACS condition, tACS was applied only for the first 60 s, and thus no tACS current flowed during rPPS. In the tACS alone condition, tACS was delivered without rPPS. (**B**) The rPPS-tACS-trough condition in the control experiment. rPPS-pulse stimulation was applied to the tACS trough phase (270° phase). (**C**) The electrode montage of tACS and TMS coil configuration. (**D**) Simulation of the electrical field induced by tACS. (**E**) Time course of the experiment. Measurements of resting motor threshold (RMT), active motor threshold (AMT), single-pulse MEPs, and paired-pulse MEPs (SICI, ICF, SAI, and SICF) were conducted before intervention. The intervention was performed for 16 min, and then RMT, single-pulse MEPs, and paired-pulse MEPs were again collected. The paired-pulse MEPs recording was divided into the two sessions, and the inter-session interval was set for 2 min. Note that test stimulus alone, SICI, ICF, SAI, and SICF were recorded in a random order before and after intervention in each participant.

For rPPS, paired stimuli (rPPS-pulse) of equal strength were applied with an ISI of 1.5 ms, every 5 s for 15 min (180 rPPS-pulse) (Fig. 1A and B)^16,20^. The stimulus intensity was set to elicit MEP amplitudes of 1 mV when delivered as a pair with an ISI of 1.5 ms.

### Transcranial alternating current stimulation

tACS was performed using a battery-driven current stimulator (DC Stimulator-Plus, NeuroConn GmbH, Ilmenau, Germany) with an intensity of 1 mA (peak-to-peak). The stimulating self-adhesive electrode (PALS electrodes, Axelgaard Manufacturing Co., Ltd., Fallbrook, CA; 3 × 3 cm) was positioned over the “hot spot” of the left M1, while a reference electrode (5 × 7 cm) was placed on the midline parietal region (Pz) (Fig. 1C). These electrode positions were adopted according to previous studies^9,15,16^. The electrical field distribution induced by tACS was simulated with SimNIBS v3.2 ^37^ (Fig. 1D). A finite element head model was derived from MRI data of one subject who was not included in this study. The stimulation waveform was sinusoidal and without DC offset, and the stimulation frequency was set at 20 Hz for β tACS. tACS was applied for 16 min, with 5 s ramp up and ramp down periods. The impedance was kept below 10 kΩ.

### Experimental design

The main experiment (Fig. 1A) consisted of three randomized conditions which were separated by at least 1 week: rPPS combined with tACS (“rPPS-tACS-peak”), rPPS during sham tACS (“rPPS-sham tACS”), and tACS alone (“tACS”). The second pulse of rPPS-pulse was adjusted to the peak phase (90°) of β tACS in the rPPS-tACS-peak condition because delivering the second pulse at I-wave periodicity was previously reported to elicit a facilitatory response^32,33^. rPPS was initiated 60 s after the beginning of the tACS, then continued for 15 min. In the sham protocol, tACS was applied for only 60 s at the beginning of the 16 min period. After the intensity determination of RMT and AMT, single-pulse 1 mV MEPs were obtained, and 20 single-pulse MEPs were recorded at rest as metrics of cortical excitability. In a paired-pulse session, TS alone, SICI, ICF, SAI, and SICF (i.e., SICF_1.5_, SICF_2.7_, and SICF_4.4_) were stimulated randomly, and eight stimuli were delivered for each parameter. Two paired-pulse TMS sessions were carried out as the baseline (“pre”); thus, 16 trials were recorded for each paired-pulse paradigm. The single-pulse MEPs and two paired-pulse sessions were separated by 2 min to avoid mental fatigue (Fig. 1E). Similar to the baseline, single-pulse MEPs and two paired-pulse sessions were recorded 5 min after intervention (“post”) because the effects immediately after intervention were not stable in our previous study^16^. For 5 min after the intervention, we measured RMT and adjusted TS intensity. Single- and paired-pulse TMS was applied with an interval ranging from 5 to 7 s to minimize any anticipatory effect. The post-measurements were recorded until approximately 23 min after intervention. In the main experiments, both the participants and the researcher who collected MEPs were blinded to the stimulation conditions (rPPS-sham tACS and rPPS-tACS-peak). To ensure the subjective experience of stimulation, participants were asked whether they perceived skin sensation and visual flickering during intervention. In addition, they were asked to look for differences between rPPS-sham tACS and rPPS-tACS-peak in the main experiment.

In addition to the main experiment, an additional control experiment was conducted. We measured the after-effects of combined stimulation in which rPPS was adjusted to β tACS at the trough phase (270°) (“rPPS-tACS-trough”, Fig. 1B). The single-pulse MEPs and two paired-pulse sessions were recorded before and 5 min after combined stimulation, and RMT was measured until 5 min after intervention. The inter-session interval was set for 2 min. TS alone MEPs, SAI, and SICF_1.5_ were recorded in the paired-pulse session. These measurements were performed to confirm the after-effects of the main experiment (rPPS-tACS-peak).

The TMS and tACS were controlled by PsychoPy^38^. Because there was a slight delay between the tACS phase and rPPS pulse, we calculated the precise rPPS pulse time accordingly, enabling us to accurately match the rPPS pulse with the tACS phase. In our experimental set up, the average time delay was 0.43 ± 0.11 ms (phase lag: 3.09° ± 0.78°), and this accuracy was similar to that reported in a previous study^16^.

### Data analysis and statistics

MEP data were visually inspected to exclude trials with background EMG activity (> 20 µV) in a 100 ms time window preceding the TMS pulse. In total, < 4% of the data were removed from single-pulse MEPs, paired-pulse MEPs, and rPPS-pulse MEPs, respectively. We measured peak-to-peak MEP amplitudes, and averaged them for each recording. Paired-pulse MEPs were calculated as a ratio to the average adjusted MEP amplitude of TS alone. To assess inter-individual variability in response to intervention, single-pulse MEPs were calculated as the ratio of post-MEPs and pre-MEPs (post/pre). Participants were arbitrarily classified as an “excitatory response” for the after-effects > 1.2 or “no response” for the after-effects < 1.2 on single-pulse MEPs^39^.

Before group comparisons, assumptions for normality were assessed using a Shapiro-Wilk test, and several MEP data sets did not follow a normal distribution. To measure the effects of the different conditions after intervention, we constructed generalized linear mixed models (GLMMs) for all MEP data analyses^40^. Models were fitted using appropriate family (gamma distributions), with identity link functions used for raw MEPs and log link functions used for the ratio of conditioned to unconditioned MEPs (i.e., paired-pulse MEPs)^40,41^. The time (pre and post) and condition (rPPS-sham tACS, rPPS-tACS-peak, and tACS) were modeled as fixed effects factors. Each model included random participant effects (intercepts and slopes)^42^, and model fit was assessed using the Akaike information criterion (AIC). However, Gaussian distribution was demonstrated for RMT data. Thus, linear mixed model (LMM) analysis was adapted to compare condition and time. We performed post hoc assessments of significant main effects and interactions using custom contrasts with the Holm-Bonferroni correction. For online effects, the rPPS-pulse MEP amplitudes during rPPS were averaged over 60 consecutive stimuli as three blocks (T1, T2, and T3), and compared among time (T1 − T3) and condition (rPPS-sham tACS and rPPS-tACS-peak) using a two-factor GLMM analysis. Finally, the contrast methods were conducted in the control experiment to compare measurements before and after intervention. The level of significance was set at *p* < 0.05. Statistical analyses were performed using JASP (version 0.19) and R^43^.

## Results

One participant reported a slight skin sensation at the beginning of the β tACS, which faded away. In the main experiment, all participants reported that they could not differentiate between rPPS-sham tACS and rPPS-tACS-peak. The physiological data in pre- and post-sessions are summarized in Table 1.

**Table 1:**
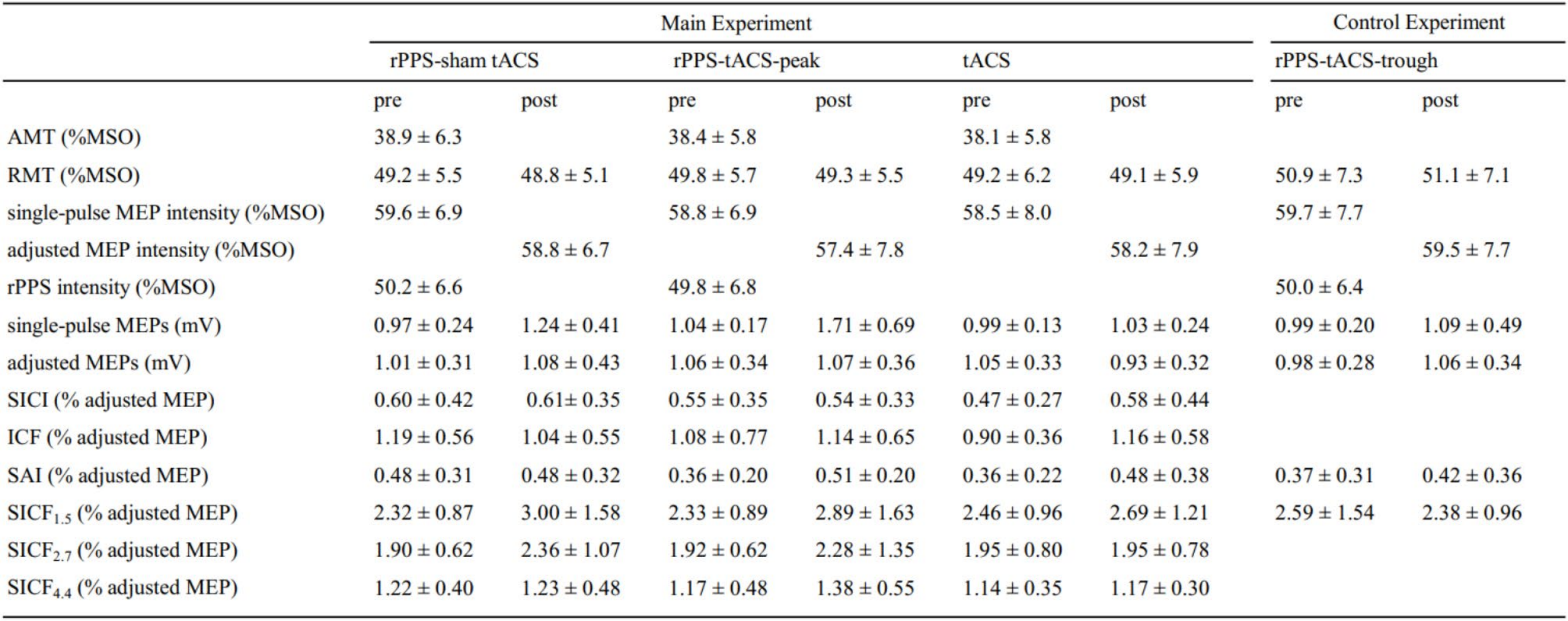
Physiological data.

Data are presented as mean ± standard deviation. %MSO, percentage of maximum stimulator output.

### Online effects of rPPS-pulse meps and their after-effects on single-pulse meps

**Fig. 2:**
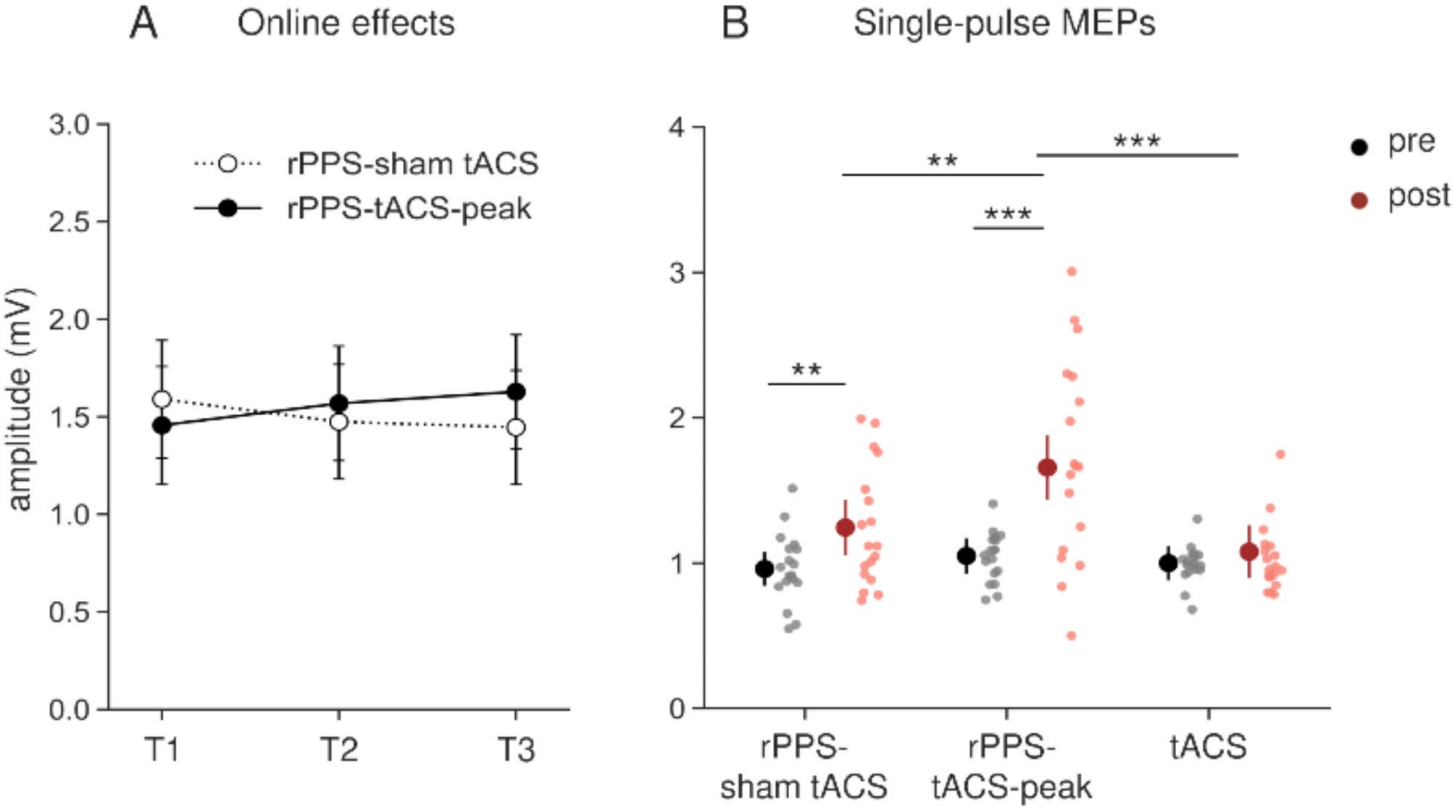
The online effects during rPPS and after-effects on single-pulse MEPs. (**A**) Amplitudes of rPPS-pulse MEPs during rPPS were averaged over 60 consecutive stimuli as three blocks (T1, T2, and T3) in the rPPS-sham tACS and rPPS-tACS-peak conditions. There was no significant difference between the two conditions. (**B**) The single-pulse MEPs in three stimulation conditions. rPPS-tACS-peak conditions increased single-pulse MEPs after intervention compared with baseline, tACS, and rPPS-sham tACS conditions. Data are presented as estimated marginal means ± 95% confidence intervals. Each dot indicates an individual participant’s after-effects on single-pulse MEPs in each condition. ** *p* < 0.01, *** *p* < 0.001.

As the online effects during rPPS intervention, there were no significant effects of time, condition, or their interaction on rPPS-pulse MEPs in GLMM (Table 2) (Fig. 2A). Figure 2B shows single-pulse MEPs in pre- and post-sessions. The rPPS-tACS-peak condition showed an increase in single-pulse MEP amplitudes compared with the rPPS-sham tACS and tACS conditions. This was confirmed by GLMM, which showed a significant interaction between time and condition (Table 2). Post hoc analysis showed a significant increase in MEP amplitudes in the rPPS-tACS-peak conditions compared with those in the rPPS-sham tACS condition and tACS (rPPS-sham tACS vs. rPPS-tACS-peak: *p* = 0.001; rPPS-tACS-peak vs. tACS: *p* < 0.001; rPPS-sham tACS vs. tACS: *p* = 0.109). Moreover, MEPs were increased after stimulation in the rPPS-sham tACS and rPPS-tACS-peak conditions compared with baseline values (rPPS-sham tACS: *p* = 0.009, rPPS-tACS-peak: *p* < 0.001, tACS: *p* = 0.398). For the RMT, LMM revealed no significant main effects of condition (*F* [2, 17] = 1.051, *p* = 0.371), time (*F* [1, 17] = 1.614, *p* = 0.221), or their interaction (*F* [2, 34] = 0.381, *p* = 0.686).

**Table 2:**
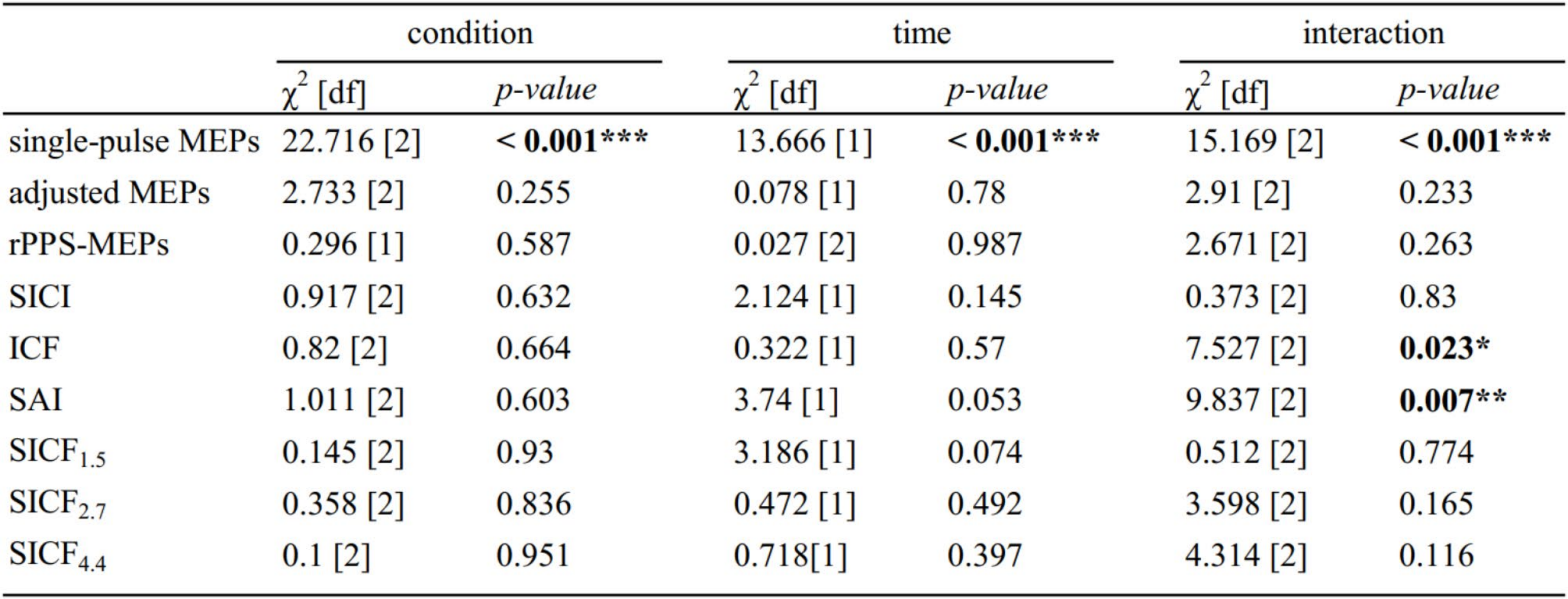
Results of generalized linear mixed model for main experiment.

*p < 0.05, ** *p* < 0.01, *** *p* < 0.001.

Regarding the inter-individual variability of after-effects on single-pulse MEPs, rPPS-tACS-peak elicited an excitatory response in most participants (excitatory response rate, rPPS-tACS-peak: 78%; rPPS-sham tACS: 67%; tACS: 17%), and these results were similar to those of a previous rPPS-tACS study (rPPS-tACS-peak: 76%; rPPS-sham tACS: 52%)^16^.

### Effects of the intervention on paired-pulse meps

**Fig. 3:**
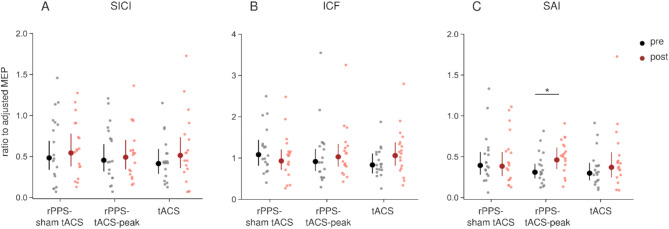
The after-effects of SICI (**A**), ICF (**B**) and SAI (**C**). Magnitudes of paired-pulse MEPs were represented as the ratio of conditioned to unconditioned MEP amplitude. There were no significant differences in SICI, ICF, and SAI among three conditions. Compared with baseline, SAI inhibition was significantly decreased in the rPPS-tACS-peak. * *p* < 0.05.

**Fig. 4:**
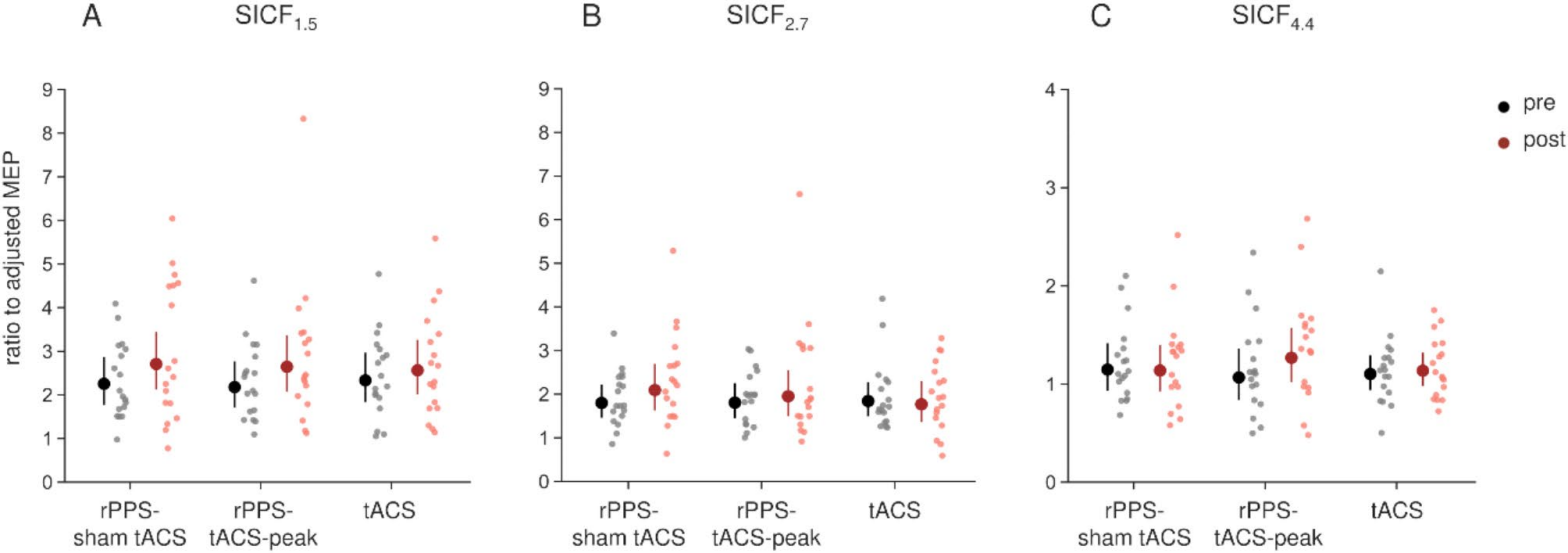
The after-effects of SICF_1.5_ (**A**), SICF_2.7_ (**B**), and SICF_4.4_ (**C**). Paired-pulse MEPs were expressed as its ratio to the adjusted MEPs. There were no significant differences in any SICF.

In the paired-pulse TMS sessions, adjusted MEPs of TS alone did not show significant main effects of time, condition, and their interaction in the GLMM (Table 2), indicating successful adjustment (Table 1). There were no significant differences among the stimulation conditions in the after-effects of SICI (Fig. 3A), as indicated by the absence of significant main effects and interactions (Table 2). In ICF, a significant interaction between condition and time was observed in the GLMM (Table 2). However, there were no significant differences between conditions or compared with baseline (*p* ≥ 0.616) (Fig. 3B). SAI was reduced compared with baseline in the rPPS-tACS-peak condition (Fig. 3C). In brief, GLMMs revealed a significant interaction of condition × time (Table 2). SAI was decreased after intervention compared with baseline in rPPS-tACS-peak only (rPPS-sham tACS: *p* = 1, rPPS-tACS-peak: *p* = 0.013, tACS: *p* = 0.547), but there were no significant differences between conditions (*p* = 1). Regarding SICF_1.5_, SICF_2.7_, and SICF_4.4_, there were no significant differences among stimulation conditions (Fig. 4). The GLMM revealed no significant main effects of condition and time, or interactions of condition × time in any SICF (Fig. 4; Table 2).

### Effects of rPPS-tACS-trough on single- and paired-pulse meps

**Fig. 5:**
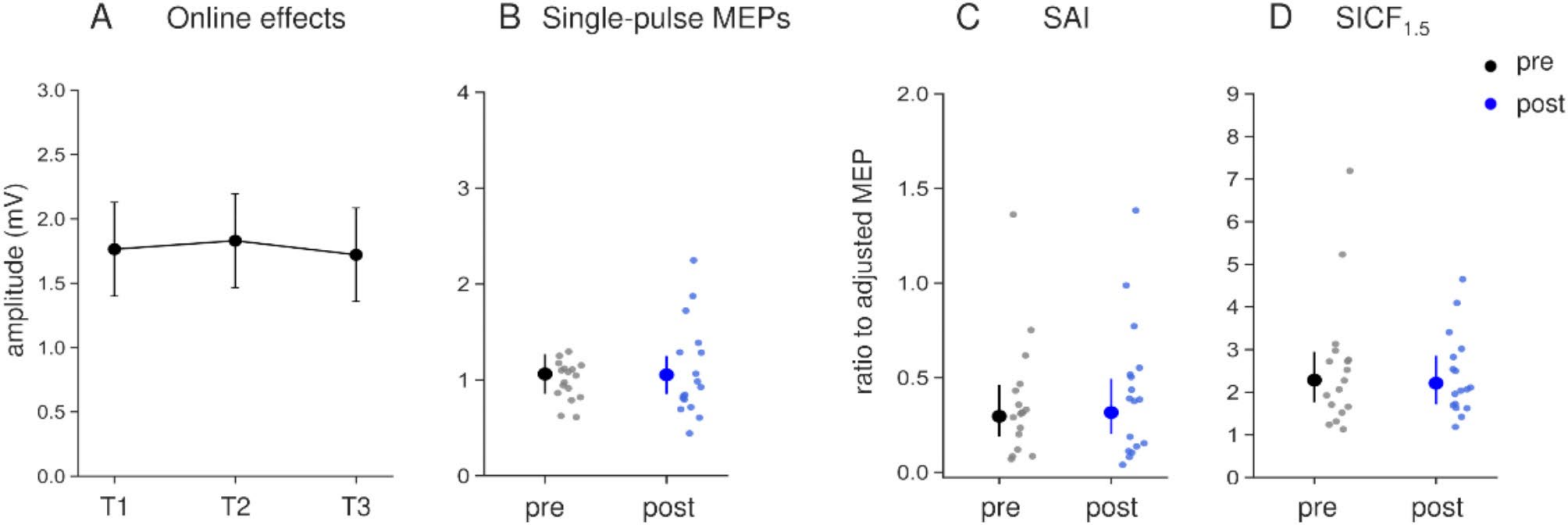
Results of rPPS-tACS-trough intervention. Amplitudes of rPPS-pulse MEPs during rPPS-tACS-trough (**A**). Amplitude of single-pulse MEPs (**B**). SAI (**C**) and SICF_1.5_ (**D**) were presented as its ratio to adjusted MEP amplitude. There were no significant differences in any measurements.

In the online effects, amplitudes of rPPS-pulse MEPs during rPPS were similar to those in the main experiment (Fig. 5A). rPPS-tACS-trough did not significantly modulate any measurements before and after intervention (Fig. 5). These results were supported by contrast methods, which showed no significant differences (single-pulse MEPs, *p* = 0.901; adjusted MEPs, *p* = 0.628; RMT, *p* = 0.591; SAI, *p* = 0.737; SICF_1.5_, *p* = 0.657).

## Discussion

We systematically investigated the mechanisms underlying the effects of rPPS-β tACS using single- and paired-pulse TMS paradigms. The present study replicated our previous results in which rPPS combined with the peak phase of β tACS (rPPS-tACS-peak) increased single-pulse MEP amplitudes after intervention. However, no significant differences existed among rPPS-tACS-peak, rPPS-sham tACS, and tACS conditions in all paired-pulse TMS measurements. Interestingly, we found that rPPS-tACS-peak protocol decreased SAI compared with baseline. The rPPS alone condition (rPPS-sham tACS) also increased single-pulse MEPs but did not significantly affect SICI, ICF, SAI, and SICF. tACS alone condition did not change any measurements. The control experiment (rPPS-tACS-trough) induced no significant modulation of single-pulse MEPs, SICF_1.5_, and SAI. Overall, our results suggest that rPPS-tACS-peak can induce plastic changes through a mechanism that is distinct from the facilitatory effect of rPPS.

### β tACS-peak does not boost rPPS by shaping I-wave facilitation

In the rPPS-tACS-peak condition, single-pulse MEPs increased compared with the baseline values and those in the rPPS-sham tACS and tACS condition. The single-pulse MEPs were enhanced in the rPPS-sham tACS condition compared with baseline, but there was no significant difference between the rPPS-sham tACS and tACS conditions. As the online effect, rPPS-pulse MEPs during rPPS were increased regardless of the stimulation conditions (rPPS-sham tACS, rPPS-tACS-peak, rPPS-tACS-trough). These results indicated that rPPS-tACS-peak induced stronger LTP-like plasticity than rPPS alone after intervention. In line with these findings, a previous study reported that rPPS-tACS-peak induced long-lasting LTP-like plasticity, which was dependent on the phase and frequency of tACS^16^. rPPS-tACS-trough induced no marked modulation of single-pulse MEPs in the control experiment. In addition, because β tACS alone left the M1 excitability unchanged, the effects of rPPS-tACS-peak cannot be explained by the simple add-on effects of β tACS on rPPS. tACS is believed to entrain cortical oscillations and enhance their power^7,8,44,45^. Thus, β tACS may boost the after-effects of rPPS by modulating β oscillations in M1.

In the SICF paradigm, paired-pulse TMS at I-wave periodicity can facilitate interactions between cortico-cortical discharges produced by the second pulse and I-waves generated by the first pulse^32,33^. SICF is thought to index the excitability within different I-wave circuits depending on ISI (i.e., 1.5, 2.7, and 4.4 ms)^33,46^. rPPS repeatedly activates facilitatory I-wave interaction, and could be considered to increase the efficacy of trans-synaptic events as spike-timing dependent plasticity^47^. Although rPPS at ISI of 1.5 ms targets earlier I-wave circuits, previous studies suggested that rPPS increased multiple I-wave peaks on SICF^19^. However, in the current study, rPPS-sham tACS did not significantly modulate SICF after intervention. This dissociation may be attributed to the variability of the data. A previous study demonstrated inter-individual variability in the response to rPPS on single-pulse MEPs^16^. Interestingly, facilitatory effects of rPPS-tACS-peak on single-pulse MEPs were more stable across the subjects (rPPS-tACS-peak, 78%; rPPS-sham tACS, 67%; tACS, 17%). This is consistent with the results of our previous study, even though different participants were tested^16^. We hypothesized that, under the rPPS-tACS-peak condition, if β tACS enhances the I-wave interaction of rPPS, differences would be observed between the stimulation conditions in both single-pulse MEPs and SICF. However, there were no significant differences in any SICF (i.e., SICF_1.5_, SICF_2.7_, and SICF_4.4_). This result suggests that the mechanism underlying the after-effect of rPPS-tACS-peak differs from that of the simple enhancement of rPPS by tACS.

What is the LTP-like plastic mechanism underlying the effects of rPPS-tACS-peak? rPPS-tACS-peak did not change RMT after intervention. The motor threshold depends on the membrane excitability of pyramidal output cells^48^, which suggests that the change in MEPs after rPPS-tACS-peak results from the modulation of trans-synaptic circuits^17^. A previous computational modeling study indicated that TMS-induced I-waves properties depend on the phase or power of ongoing brain oscillations^49^. A TMS-EEG study revealed that β oscillation in the motor cortex modulated MEP amplitude or latency in a specific phase^50^. These findings suggest that the optimal phase of cortical β rhythm in M1 may represent windows of raised cortical excitability^50^. In addition, the phase of tACS is considered to affect neural spike timing^6,8^, and β tACS modulated M1 cortical excitability in a phase-dependent manner^11,12^. Phase-dependent plasticity provides one possible explanation of the after-effects of rPPS-tACS-peak. When the excitatory postsynaptic potential (EPSP) coincided with the peak phase of membrane potential oscillations imposed by alternating current fields in the β range in the rat visual cortex, LTP was induced in a phase-dependent manner^51^. In this scenario, combined stimulation, in which EPSP generated by rPPS-pulse is adjusted to the rising excitatory window induced by β tACS, leads to an increase in single-pulse MEPs after stimulation. The results of rPPS-tACS-trough also support this hypothesis. An alternative explanation is that rPPS-tACS-peak can boost the different cortical networks from the I-wave circuits. A previous study reported that rPPS significantly increased MEP amplitudes, but was accompanied by only a slight increase in the amplitude of I-waves in epidural recordings^52^. As a reason for the lack of evidence in the epidural recordings, the authors suggested that rPPS can facilitate long-range connections originating from remote areas, evoking more dispersed additional activity^52,53^.

### After-effects of rPPS-tACS on SAI, SICI, and ICF

In the present study, although SAI decreased in the rPPS-tACS-peak compared with baseline after stimulation, no significant differences were observed between stimulation conditions. SAI is thought to contribute to neural interactions within the cerebral cortex, and to reflect inhibitory somatosensory projections to the M1 ^28^. Several studies proposed that β oscillation modulation in the motor cortex is involved in sensory reafferences, playing an inhibitory role in M1^54,55^. Moreover, the SAI paradigm could induce the attenuation of the amplitude of TMS-evoked potential and β rhythm selective decrement of phase locking in the motor cortex in addition to the inhibition of MEP amplitudes^56^. These results suggest a possible inverse relationship between β oscillations in M1 and SAI inhibitory activity. Previous studies indicated that β tACS decreased SAI during stimulation (online effect) as causal evidence^11,21^. Contrary to this online effect, we found that β tACS alone did not alter SAI after intervention. These results suggest that β tACS may influence the SAI network during but not after stimulation. Interestingly, although rPPS-tACS-peak did not modulate SAI compared with other stimulation conditions, it showed a significant decrease relative to baseline. Therefore, the mechanisms of LTP-like plasticity induced by rPPS-tACS-peak may partially involve a decrease in cholinergic inhibition in sensorimotor integration during β tACS. It is possible that this online effect of β tACS was enhanced by combined stimulation, leading to the small but observable after-effect on SAI.

Previous studies also revealed that β tACS with low intensity (≤ 1 mA) did not produce after-effects on single-pulse MEPs^57,58^, SICI, and ICF^57^. These findings suggest that 1 mA β tACS cannot modulate intracortical GABAa inhibitory and glutamatergic facilitator interneurons in a way that is captured by TMS. However, β oscillations in the sensorimotor cortex have been suggested as a link to GABAergic inhibition^59,60^. Interestingly, pharmacological studies have shown that SAI is mediated via not only cholinergic neurons^30^ but also the activity of GABAergic neurons^61,62^. Moreover, a previous study showed the dissociating patterns of GABAergic drug modulation of SICI versus SAI, and suggested that different GABA receptor subtypes are involved in SAI and SICI^63^. Therefore, it is possible that changes in GABAergic modulation by β tACS could be assessed using paired-pulse TMS paradigms other than SICI. Additionally, it may be necessary to increase the intensity of tACS to clarify the after-effects on SICI, ICF, and single-pulse MEPs, as suggested by a previous study reporting that 2 mA β tACS induced NMDA-mediated plasticity, and single-pulse MEPs were increased after stimulation^64^.

### Limitations

This study involved several limitations. First, we did not directly assess the modulation of β oscillations in M1, which could be affected by combined stimulation. In the visual cortex, α tACS increased both the power of α oscillation and the amplitude of visual evoked potentials after stimulation^45^. Thus, the use of electroencephalography may contribute to further clarification of the relationship between β oscillation and LTP-like plasticity by combined stimulation. Second, the present study involved the measurements of paired-pulse TMS until approximately 23 min after stimulation in the main experiment. A previous study revealed that the facilitatory effects of combined stimulation persisted for over 30 min after stimulation, although single-pulse MEPs were enhanced for only approximately 10 min after rPPS alone^16^. Thus, the timing of paired stimulation measurements in this study may have been late for rPPS alone. However, a previous study revealed that rPPS increased multiple SICF peaks until 15–20 min after stimulation^19^. Therefore, the effects of rPPS on SICF may be longer compared with single-pulse MEP modulation. Finally, participants in the current study may have been able to identify the tACS condition because of the lack of rPPS. Thus, we cannot completely rule out the possibility that a placebo effect may have occurred in the tACS condition, although the rPPS-tACS-peak and rPPS-sham tACS conditions were blinded to participants and the TMS investigator.

## Conclusions

We explored the mechanisms underlying the after-effects of combined stimulation with rPPS and β tACS at peak phase using paired-pulse TMS paradigms. rPPS-tACS-peak increased M1 excitability compared with rPPS and tACS alone. However, combined stimulation did not significantly modulate SICF. Thus, this facilitation effect cannot be attributed to enhanced I-wave interactions induced by rPPS. However, we found a decrease in SAI in rPPS-tACS-peak after intervention without significant differences between conditions. This finding suggests that the effects of rPPS-tACS-peak may be partially mediated by cholinergic inhibition in sensorimotor integration. Our results provide new insights for understanding the mechanisms underlying combined stimulation using tACS and could potentially contribute to the development of therapeutic applications using combined stimulation.

## Data Availability

The datasets used and/or analyzed during the current study are available from the corresponding author on reasonable request.
